# Molecular Characterization and Epidemiology of Carbapenem-Resistant *Enterobacteriaceae* Isolated from Pediatric Patients in Guangzhou, Southern China

**DOI:** 10.1155/2023/4762143

**Published:** 2023-01-30

**Authors:** Fei Gao, Zhile Xiong, Bingshao Liang, Zhenting Huang, Qiulian Deng, Jielin Wang, Huamin Zhong, Yan Long, Sufei Zhu

**Affiliations:** Clinical Laboratory, Guangzhou Women and Children's Medical Center, Guangzhou Medical University, Guangzhou, Guangdong 510623, China

## Abstract

**Background:**

Carbapenem-resistant *Enterobacteriaceae* (CRE) are spreading worldwide, posing a serious public health concern. However, the data on CRE strains that cause infections in children in Guangzhou remain limited. Therefore, this study aimed to investigate the epidemiology of CRE, drug resistance, and resistance mechanisms in children in Guangzhou, Southern China.

**Methods:**

In total, 54 nonrepetitive CRE strains were collected in pediatric patients at three centers in Guangzhou, Southern China, from January 2016 to August 2018. CRE isolates were used for further studies on antimicrobial susceptibility, the modified Hodge test (MHT), the modified carbapenem inactivation method (mCIM), and drug resistance genes. Multilocus sequence typing (MLST) was used to elucidate the molecular epidemiology of *K. pneumoniae*.

**Results:**

The isolated CRE strains include 34 *K. pneumoniae* (63.0%), 10 *E. coli* (18.5%), 4 *Enterobacter cloacae* (7.4%), and 6 *Proteus mirabilis* (11.1%) strains. The strains were isolated mainly from the blood (31.5%, *n* = 17), sputum (31.5%, *n* = 17), and urine (16.7%, *n* = 9). All CRE isolates were highly resistant to the third- or fourth-generation cephalosporins, carbapenems, and *β*-lactam + *β*-lactamase inhibitors (94.4%–96.3%). In addition, the resistance rates to amikacin, ciprofloxacin, levofloxacin, tigecycline, and colistin were 5.6%, 14.8%, 16.7%, 9.3%, and 0%, respectively. Carbapenemase was detected in 35 (64.8%) of the CRE isolates. The most dominant carbapenemase gene was *bla*_*NDM*_ (*n* = 17, 48.6%), followed by *bla*_*IMP*_ (*n* = 13, 37.1%) and *bla*_*OXA*-23_ (*n* = 4, 11.4%). Other carbapenemase genes (*bla*_KPC_, *bla*_sim_, *bla*_Aim_, *bla*_GES_, *bla*_Gim_, *bla*_*OXA-2*_, and *bla*_*OXA-48*_) and the *mcr*-1 gene were not detected. MLST analysis showed high diversity among the *K. pneumoniae*, and ST45 (11.7%, 4/34) was the dominant sequence type.

**Conclusion:**

This study revealed *bla*_NDM_ was the most dominant carbapenemase gene in children in Guangzhou. Infection control measures need to be taken for the prevention and treatment of CRE infections.

## 1. Introduction

*Enterobacteriaceae*, represented by *Klebsiella pneumoniae* and *Escherichia coli*, are considered as one of the most important pathogenic bacteria for hospital infections in recent decades [1, 2]. Carbapenems were deemed the last resort for the treatment of Gram-negative bacteria (GNB). Unfortunately, the emergency of carbapenem-resistance *Enterobacteriaceae* (CRE) has become an independent risk factor for the death of patients with nosocomial infection. Drug resistance to carbapenems has led to difficulties in treating many critically ill patients with GNB infections in clinical practice [3, 4]. Therefore, much attention needs to be paid to CRE infections in the fields of epidemiology, antimicrobial therapy, and drug-resistant gene detection.

The mechanism of CRE is predominantly attributed to the production of carbapenemases, including class A (*bla*_KPC_), class B (*bla*_NDM,_*bla*_IMP_, and *bla*_VIM_), and class D (*bla*_OXA-23_ and *bla*_OXA−48_) of the Amble systems [5, 6]. Several studies have reported that the rapid spread of CRE in the community and the nosocomial area is largely attributed to the dissemination of carbapenemases, which may lead to hospital outbreaks and become endemic in healthcare areas [7, 8]. In addition, carbapenemase genes could co-exist with extended-spectrum *β*-lactamases (ESBLs) on plasmids which would further limit the treatment options for patients. It has been reported that sequence type (ST) 258 has significantly contributed to the dissemination of *K. pneumoniae* harboring *bla*_KPC_ in adults in the United States [9] and ST11 is the predominant type in China [10]. However, the molecular epidemiology of CRE in children in Southern China remains unknown.

Due to imperfections in organ development, children more easily suffer from bacterial infections and are sensitive to the side effects of drugs. The correct approach in the treatment of antibiotics should be adjusted in time after diagnosis, and narrow-spectrum and low-toxicity drugs should be used for patient treatment. Several studies have indicated that the genotype and drug resistance spectrum of carbapenemases are different between regions and hospitals [11, 12]. Therefore, there is an urgent need to investigate the drug resistance mechanisms and molecular epidemiology of CRE in children in Southern China. To address this challenge, we aimed to investigate the epidemiology of CRE, drug resistance, and resistance mechanisms in children in three medical centers in Guangzhou, Southern China.

## 2. Materials and Methods

### 2.1. Bacterial Isolate Collection

From January 2016 to August 2018, 54 unduplicated clinical CRE isolates were obtained from three centers in Guangzhou, as previously described [13]. Samples were collected from different clinical specimens (blood, urine, sputum, pleural effusion, and catheters). CRE were defined as the strains that are resistant to one of the three carbapenems (imipenem, ertapenem, or meropenem) [14]. All isolates were identified using an automated VITEK MS (bioMérieux, Marcy l'Étoile, France).

### 2.2. Antimicrobial Susceptibility Testing

Antibiotic susceptibility for 16 antibiotics (cefoperazone/sulbactam, piperacillin/tazobactam, ceftazidime, cefepime, aztreonam, imipenem, meropenem, amikacin, tobramycin, levofloxacin, ciprofloxacin, trimethoprim/sulfamethoxazole, doxycycline, tetracycline, minocycline, and colistin) was detected using the automated VITEK2 compact system (bioMérieux) with VITEK 2 AST-N335 cards (bioMérieux). The results were interpreted according to the guidelines of the Clinical and Laboratory Standards Institute (CLSI) in 2019. The strains for quality control of antibiotic susceptibility tests were E. coli standard strain ATCC25922 and K. pneumoniae ATCC700603.

### 2.3. Detection of Antimicrobial Resistance Genes

Polymerase chain reaction (PCR) was used to detect carbapenemase genes (*bla*_*KPC*_*, bla*_*sim*_*, bla*_*Aim*_*, bla*_*GES*_*, bla*_*NDM*_*, bla*_*IMP*_*, bla*_*VIM*_*, bla*_*Gim,*_*bl*_*OXA-2*_*, bla*_*OXA-10*_*, bla*_*OXA-23*_, and *bla*_*OXA-48*_), ESBL genes (*bla*_*SHV*_*, bla*_*TEM*_, and *bla*_*CTX-M*_), and the mcr-1 gene in all CRE strains [15, 16]. PCR was performed according to a previous study. [13] The modified Hodge test (MHT) and modified carbapenem inactivation method (mCIM) were used to confirm the production of carbapenemases [17].

### 2.4. Multilocus Sequence Typing

Multilocus sequence typing (MLST) of K. pneumoniae was performed according to the protocol described on the website (https://www.pasteur.fr/recherche/genopole/PF8/mlst/Kpneumoniae.html). Seven housekeeping genes of K. pneumoniae (gapA, ropB, mdh, infB, pgi, phoE, and tonB) were amplified and sequenced according to a previous description [18]. Then, the sequence types (STs) were determined by comparing the sequences in the MLST database. All 34 *K. pneumoniae* isolates were clustered based on the MLST database using the minimum spanning tree method in BioNumerics software (Applied Maths, Sint-Martens-Latem, Belgium).

### 2.5. Statistical Analysis

Statistical analyses were performed using SPSS software 22 (SPSS Inc., Chicago, IL, USA). Quantitative data were compared using the *t*-test, and categorical data were evaluated using the *χ*^2^ test or Fisher's exact test. *p* < 0.05 was considered a statistically significant difference.

## 3. Results

### 3.1. Clinical and Epidemiological Characteristics

A total of 54 CRE were collected from various clinical specimens from January 2016 to August 2018 in a pediatric patient in Guangzhou, Southern China. Of the CRE strains, 34 *K. pneumoniae* (63.0%), 10 *E. coli* (18.5%), 4 *Enterobacter cloacae* (7.4%), and 6 *Proteus mirabilis* (11.1%) strains were isolated (Table 1).

**Figure 1:**
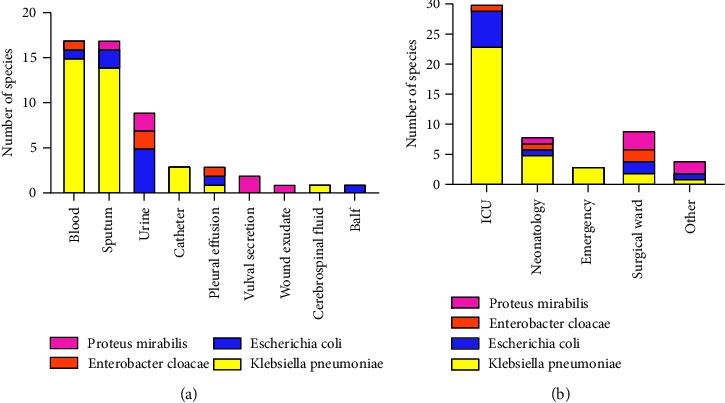
(a) Distribution of CRE strains isolated from different samples and (b) the distribution of CRE strains in different departments. ICU, intensive care unit.

The CRE strains were obtained from blood (31.5%, *n* = 17) and sputum (31.5%, *n* = 17), followed by urine (16.7%, *n* = 9), catheter (5.6%, *n* = 3), pleural and peritoneal effusions (5.6%, *n* = 3), vulval secretion (3.7%, *n* = 2), wound exudate (1.9%, *n* = 1), cerebrospinal fluid (1.9%, *n* = 1), and alveolar lavage fluid (1.9%, *n* = 1). The specimen's samples were sent from the intensive care unit (57.4%, *n* = 31), followed by the neonatal unit (13.0%, *n* = 7), surgical unit (14.8%, *n* = 8), emergency department (5.6%, *n* = 3), and other areas (9.3%, *n* = 5) (Figure 1).

### 3.2. Antimicrobial Susceptibility Tests

As shown in Table 1, all CRE isolates showed high resistance to carbapenemases, *β*-lactam+*β*-lactamase inhibitors, third- or fourth-generation cephalosporins (94.4%–96.3%), minocycline (85.2%), doxycycline (77.8%), and aztreonam (72.2%). The resistance rates to amikacin, ciprofloxacin, levofloxacin, tigecycline, and colistin were 5.6%, 14.8%, 16.7%, 9.3%, and 0%, respectively. A portion of the *bla*_*NDM*_-producing CRE isolates showed resistance to tobramycin (11.8%) and doxycycline (94.1%). However, the opposite result was observed among *bla*_*IMP*_-carrying strains (both 53.8%) (*p* < 0.05). All the CRE isolates were categorized as multidrug-resistant bacteria (MDR).

### 3.3. Carbapenemase and Other Resistance Genes

The prevalence and molecular characterization of different carbapenemase and ESBL genes among the 54 CRE strains are shown in Table 2. Among the 54 strains, 35 were confirmed as positive for carbapenemases according to the mCIM and MHT tests. Twelve *K. pneumoniae*, four *E. coli*, one *E. cloacae*, and one *Proteus mirabilis* strains were detected to produce carbapenemase and were resistant to only one or two of the tested carbapenem. *bla*_*NDM*_ (48.6%, 17/35) was the most prevalent carbapenemase gene, detected in 11 *K. pneumoniae*, five *E. coli*, and one *E. cloacae* strains, followed by *bla*_*IMP*_ (34.3%, 12/35), *bla*_*OXA*-23_ (11.4%, 4/35), *bla*_*OXA*-10_ (2.9%, 1/35), and *bla*_*VIM*_ (2.9%, 1/35). Besides, none of the *bla*_*KPC*_*, blasim, bla*_*GES*_, or *mcr*-1 genes were detected. Meanwhile, the ESBL genes SHV (59.3%, 32/54), TEM (44.4%, 24/54), and CTX-M (33.3%, 18/54) were also detected. Fourteen CRE strains carried both TEM and SHV genotypes.

### 3.4. Clinical Characteristics and Clonal Relatedness of Carbapenem-Resistant *K. pneumoniae* (CRKP)

**Figure 2:**
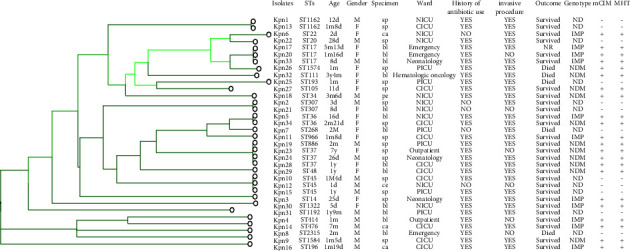
Dendrogram of MLST data from the *K. pneumoniae* isolates. The UPGMA algorithm was performed to construct a dendrogram based on the dice similarity coefficient. KPN, *K. pneumoniae*; d, day; m, month; y, year; M, male; F, female; sp, sputum; ca, catheter; bl, blood; pe, pleural effusion; PICU, pediatric intensive care unit; NICU, neonatal intensive care unit; CICU, cardiac intensive care unit.

**Figure 3:**
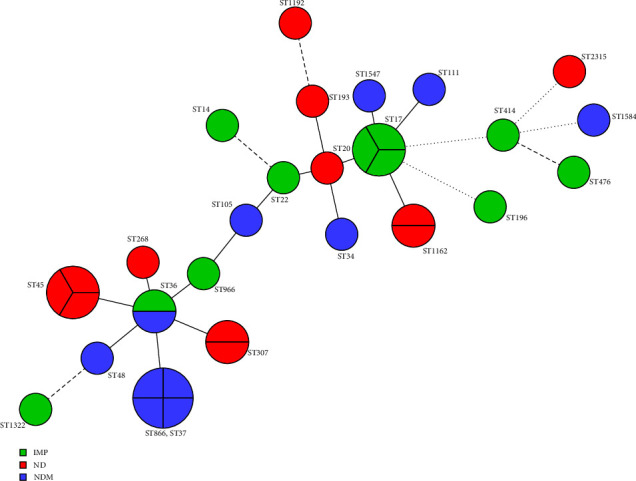
Minimum spanning trees of 34 CRKP isolates. Each node represents a single ST. The size of the nodes is proportional to the number of strains within the representative ST. The color distribution represents the distribution of carbapenemase genes among 24 different STs.

As shown in Figure 2, MLST analysis revealed 23 different STs among the 34 CRKP isolates. ST45 (11.7%, 4/34) was the dominant type, followed by ST17 (8.82%, 3/34), ST37 (8.82%, 3/34), ST36 (5.88%, 2/34), ST307 (5.88%, 2/34), ST1162 (5.88%, 2/34), and ST414 (5.88%, 2/34). Others include ST20, ST22, ST34, ST45, ST48, ST105, ST111, ST193, ST196, ST866, ST966, ST1192, ST1322, ST1547, ST1584, and ST2315. The distribution of carbapenemases among the different STs in *K. pneumoniae* is shown in Figure 3. For *K. pneumoniae*, *bla*_*NDM*_ was detected in ST37, ST48, ST105, ST111, and ST1584, whereas bla_IMP_ was detected in ST14, ST17, ST22, ST36, ST196, ST414, ST476, ST966, and ST1322.

The clinical characteristics of CRKP patients are shown in Figure 2. More than 75% of CRKP cases have a history of antibiotic exposure and invasive procedures. Five patients died during the CRKP infections. Moreover, CRKP is mainly distributed in ICU (73.53%, 25/34), which includes CICU (29.41%, 10/34), NICU (26.47%, 9/34), and PICU (17.65%, 6/34).

## 4. Discussion

Carbapenems such as meropenem and imipenem are considered the last line of defense against serious infections caused by *Enterobacteriaceae* [19, 20]. With the widespread use of carbapenems in nosocomial areas, the detection rate of CRE has been increasing annually, particularly in CRKP [21, 22]. According to a multicenter study conducted from 2013 to 2014 in the United States, the carbapenem-resistant *Enterobacteriaceae* detection rate rapidly increased from 5% to approximately 10% [6]. The CHINET study showed that the number of CRE strains increased from 5.82% in 2011 to 30% in 2016, an average increase of 23.1% [4].

CRE is an emerging problem that spreads among children, one of the most vulnerable populations [23]. Guidelines for antibiotic use in children with CRE infection need to be developed. In this study, we investigated drug resistance in CRE isolated from children in Guangzhou. Antimicrobial susceptibility tests showed that all 54 CRE strains were highly resistant to *β*-lactamases, carbapenems, and third- and fourth-generation cephalosporins. However, antimicrobial susceptibility tests showed that 54 CRE had low resistance to quinolones and aminoglycoside antibiotics, such as amikacin, levofloxacin, and ciprofloxacin. This may be due to the preference for *β*-lactamase antibiotics for reinfection in pediatrics, which results in more ESBLs producing clinical strains. In addition, the reason for CRE's high resistance to third- or fourth-generation cephalosporins may be due to biofilm formation among *Enterobacteriaceae* [24–26]. With the widespread use of carbapenems instead of *β*-lactamases for pediatric treatment, the number of CRE will increase. According to this study, quinolones and aminoglycoside antibiotics may be effective against CRE.

Studies had shown that the resistance mechanism of CRE clinical strains was isolated from pediatric patients in Guangzhou, Southern China. *bla*_NDM_ was the most dominant genotype in the pediatric patient population in Guangzhou, Southern, China. Interestingly, no *bla*_KPC_ gene was detected in this study, and *bla*_KPC-2_ was mostly prevalent in the adult population, which means that the strategy for anti-CRE treatment for children would be different from that for adult.

In this study, the ICU (NICU, PICU, and CICU) was the most common department with cases of CRE infection in our medical center, which might be closely related to critical underlying diseases, long-term application of carbapenems and other antimicrobial agents, and prolonged hospitalization in the ICU. This is consistent with a previous study in which ICUs were known reservoirs for multidrug-resistant bacteria in healthcare settings [27]. This suggests that correlated measures are needed to curtail CRE spread in the ICU. In addition, a large proportion of CRE strains in our study were isolated from sputum and blood specimens, which suggests that respiratory tract colonization had occurred and admission to the ICU may be a risk factor for CRE bacteremia. However, these observations need to be confirmed by further studies using a larger sample size and appropriate adjustments for confounding factors.

In this study, *K. pneumoniae* was the dominant CRE clinical isolate, which is consistent with other clinical CRE studies. [28, 29] In China, the most dominant ST type in *K. pneumoniae* is ST11, which carries KPC. [30] However, no ST11 was detected in this study, suggesting that ST11 is not prevalent in children in Southern China. In this study, MLST showed high diversity among CRKP isolates, with 24 diverse ST types. ST45 was the first ST type in this study. Interestingly, ST45 *K. pneumoniae* carries no carbapenemase, which means that these strains would not cause severe spread in the clinical setting. ST17 and ST37 were the second- and third-most common ST types, respectively. ST17 carries *bla*_*IMP*_, and ST37 carries *bla*_*NDM*._ A previous study reported that ST17 belonged to the hypervirulent CC17 lineage [31]. In the presence of ST17 *K. pneumoniae* carrying bla_IMP,_ some corresponding measurements need to be implemented. In addition, the virulence of *K. pneumoniae* still needs further study. The MLST results also indicated that the CRKP in the hospital is not due to the expansion of clonal lineages but has emerged from multiple sources.

With the emergence of CRE, optimization of detection technology is a challenge for microbial laboratory diagnostics [32]. A previous study showed that molecular biological methods for carbapenemase gene detection are the gold standard [33]. However, false-negative results (mutations or unexpressed genes) have limited their clinical use. CIM is a method with high sensitivity, high specificity, and low cost, and mCIM was included in the 2017 detection method of carbapenemase production in CLSI [34]. In this study, the performance and characteristics of the Hodge test and the mCIM test were compared by detecting 54 clinical carbapenem-resistant strains. Thirty-five strains producing carbapenemase were detected by the mCIM test after PCR results and sequence analysis, whereas two strains of NDM carbapenemase were not detected by the modified Hodge test. This may be due to other resistance mechanisms, such as AmpC production and/or ESBL production combined with reduced permeability. A previous study showed that the sensitivity and specificity of mCIM for carbapenemase-producing *Enterobacteriaceae* (CPE) detection were generally above 97% [35, 36]. A study by Yamada and her colleague [37] revealed that, compared with the detection of carbapenemase strains by the mCIM and MHT, results showed false-negative results in the detection of NDM, but its sensitivity (98.8%) and negative predictive value (99%) were higher. mCIM is an accurate method for detecting carbapenemases. The only drawback is that overnight cultures are required to produce results.

This study had some limitations. First, it was a retrospective study with a relatively small study population of children. Second, information on clinical characteristics and outcomes could not be completely acquired. Third, we did not perform a risk factor analysis between CRE infection and carbapenem-susceptible *Enterobacteriaceae* in children in Southern China; therefore, infections for which cultures were not obtained were missed. Thus, further studies and additional experiments are required.

## 5. Conclusion

This study revealed that *K. pneumoniae* and *E. coli* were the main CRE isolated from Guangzhou Women's and Children's Medical Center. The ESBL genotypes carried by CRE in our hospital were mainly TEM and SHV, and the genotypes of carbapenemase were mainly *bla*_*NDM*_ and *bla*_*IMP*._ There was no *bla*_*KPC*_ was found. Therefore, early detection of bacteria for nosocomial infection control is an important measure to prevent infection and transmission of CRE strains.

## Figures and Tables

**Table 1:** In vitro activities of antimicrobial agents against CRE.

Antimicrobials	All isolates (*n* = 54)			*bla*_*NDM*_ + strains (*n* = 17)			*bla*_*IMP*_ + strains (*n* = 13)			Comparison between NDM and IMP	
	*R*(%)	MIC50	MIC90	*R*(%)	MIC50	MIC90	*R*(%)	MIC50	MIC90	*χ* ^2^	*p*value
Piperacillin/tazobactam	94.4	≥128	≥128	100	≥128	≥128	92.3	≥128	≥128	—	0.245
Cefoperazone/sulbactam	94.4	≥64	≥64	100	≥64	≥64	92.3	≥64	≥64	—	—
Ceftazidime	94.4	≥64	≥64	100	≥64	≥64	76.9	≥64	≥64	—	—
Cefepime	94.4	≥32	≥32	100	≥32	≥32	76.9	≥32	≥32	—	
Aztreonam	72.2	≥64	≥64	76.5	≥64	≥64	69.2	≥64	≥64		
Imipenem	96.3	≥16	≥16	100	≥16	≥16	100	≥16	≥16	—	—
Meropenem	94.4	≥16	≥16	100	≥16	≥16	84.6	≥16	≥16	—	—
Tobramycin	29.6	≤1	8	11.8	≤1	8	53.8	8	8	6.212	0.013
Amikacin	5.6	≤2	≤2	0	≤2	≤2	0	≤2	≤2	—	—
Levoacinine	16.7	1	1	11.8	≤0.12	≥8	0	1	4	—	—
Ciprocininen	14.8	0.5	1	11.8	≤0.25	≥4	7.7	0.5	1	—	—
Sulfamethoxazole/trimethoprim	33.3	2/38	≥16/30	70.6	≥16/30	≥16/30	46.2	≤1/19	≥16/30	3.096	0.078
Colistin	0	≤0.5	≤0.5	0	≤0.5	≤0.5	0	≤0.5	≤0.5	—	—
Doxycycline	77.8	≥16	≥16	94.1	≥16	≥16	53.8	≥16	≥16	6.679	0.01
Minocycline	85.2	≥16	≥16	94.1	≥16	≥16	69.2	8	≥16	3.285	0.07
Tigecycline	9.3	1	≥8	0	1	2	15.4	1	2	—	—

**Table 2:** Prevalence of different carbapenemase and ESBL genes among 54 CRE strains.

	Numbers (%)	Carbapenemase phenotypic confirmatory test positive (*n*)	Carbapenemase					ESBL		
			bla NDM	bla IMP	bla VIM	bla OXA10	bla OXA23	TEM	SHV	CTX-M
*K. pneumoniae*	34 (62.96)	23	11	12	—	—	—	16	27	14
*E. coli*	10 (18.52)	6	5	1	—	—	—	7	1	3
*Enterobacter cloacae*	4 (7.41)	2	1	—	1	1	—	1	2	1
*Proteus mirabilis*	6 (11.11)	4	—	—	—	—	4	—	—	
Total	54 (100)	35	17	13	1	1	4	24	30	16

## Data Availability

The data generated or analyzed during this study are included within the article.
